# Design and Experiment of a Biomimetic Duckbill-like Vibration Chain for Physical Weed Control during the Rice Tillering Stage

**DOI:** 10.3390/biomimetics8050430

**Published:** 2023-09-18

**Authors:** Longyu Fang, Xiwen Luo, Zaiman Wang, Wenwu Yang, Hui Li, Shiyu Song, Haoyang Xie, Jianhao Hu, Weiman Chen, Qinghai Liu

**Affiliations:** 1 College of Biological and Agricultural Engineering, Jilin University, Changchun 130022, China; 2 Key Laboratory of Key Technology on Agricultural Machine and Equipment, Ministry of Education, South China Agricultural University, Guangzhou 510642, China; 3 Huangpu Innovation Research Institute, South China Agricultural University, Guangzhou 510725, China; 4 Hunan Academy of Agricultural Sciences, Changsha 410125, China

**Keywords:** biomimetic duckbill-like, vibration chain, physical weed control in paddy fields, tillering stage

## Abstract

The widespread use of chemical herbicides has jeopardized concerns about food safety and ecological consequences. To address these issues and reduce reliance on chemical herbicides, a physical weed control device was developed for the tillering stage in paddy fields. This device features a biomimetic duckbill-like vibration chain that effectively controls weed outbreaks. The chain penetrates the soft surface soil of the paddy field under gravity and rapidly stirs the soil through vibration, leading to the detachment of the weed roots anchored in the surface layer. Simultaneously, the device avoids mechanical damage to rice seedlings rooted in deeper soil. This study aimed to investigate the effects of chain structural parameters (the number of chain rows, vibration amplitude, and length of chains) and operational parameters (vibration frequency and working velocity) on weed control efficiency and rice seedling damage. Through a central composite regression field test, the optimal device structure and operational parameters were determined. The optimization results demonstrated that a vibration amplitude of 78.8 mm, a chain length of 93.47 cm, and 3.4 rows of chains, along with a vibration frequency and working velocity ranging from 0.5 to 1.25 m/s, achieved an optimal weeding effect. Under the optimal parameter combination, field test results demonstrated that approximately 80% of the weeds in the field were effectively cleared. This indicates that the design of the biomimetic duckbill-like vibration chain weeding device exhibits a relatively superior weeding performance, offering a practical solution for the management of weeds in rice fields.

## 1. Introduction

The growth of rice crops is highly sensitive to weed competition in the early stages. Research conducted by Shrivastava et al. has shown that failure to adhere to proper crop management practices can result in grain yield losses of 20–30% depending on weed density [1]. If weeds are not controlled within the first three weeks after sowing (the tillering stage), the yield can be reduced by 50% [2,3]. Research has shown that weed control in paddy fields during the tillering stage not only achieves the highest weed removal rates but also attains optimal economic efficiency [4,5]. Thus, early weed control and removal are essential [6].

Although methods such as crop rotation, biological control, and straw mulching can effectively control weed growth, herbicide use remains the primary means of weed control [7]. However, the long-term use of herbicides can lead to weed resistance, reduced herbicide efficacy, and serious threats to agroecosystems and food safety, which ultimately endangers human health and survival [8,9,10]. Glyphosate, a commonly used contact herbicide, has been linked to cancer and can leave residues in crops such as flour, rice, and onions, as well as in animals, such as cows, fish, and domestic pets [11,12,13,14,15]. Paraquat, another widely used herbicide, poses a great danger to humans upon contact and threatens the safety of pesticide applicators, while crop losses due to pesticide drift have been reported [16,17]. Therefore, there is a growing demand for physical weed control methods to replace chemical weed control.

Mechanical weeding offers a safer and more reliable solution to weed management in paddy fields. Research by Maimunah et al. has shown that mechanical weeding not only reduces the chance of weed outbreaks but also significantly improves nitrogen uptake and agronomic utilization efficiency in rice, resulting in increased yields [18]. Various mechanical weed control methods have been developed, including the weeding turtle robot designed by Nakamura et al. [19]. Tian et al. designed a self-propelled paddy field weeding machine and conducted dynamic modeling research on the interaction between the walking wheel and soil, providing support for the motion model of the paddy field walking chassis [6]. Tang et al. designed a weeding wheel with rake teeth to bury the weeds between the rice rows [20]. Jiao et al. designed a weeding roller with spirals to press the weeds between the rice rows into the soil, achieving the same effect as chemical weeding [21]. However, these weeding methods inevitably have issues such as damaging rice seedlings and crushing them. Moreover, it is necessary to develop different weeding devices for weeds between the rice rows and within the rows. A more gentle and effective weeding method is urgently needed to clear weeds in the fields with minimal damage to rice seedlings.

Research has found that wild animals living in paddy fields can have potential benefits for rice production [22,23]. Their movements in the water can increase turbidity, blocking sunlight and inhibiting weed growth [24]. This biological disturbance can also increase the oxygen content in the soil, promote microbial activity, and stimulate root growth, resulting in improved crop growth and yield [25]. Therefore, it is necessary to develop a more ecologically friendly and effective weeding method that considers the relationship between rice crops and wild animals in paddy fields.

The symbiotic system of ducks and rice crops has a long history and has been recognized as an effective way to suppress weeds in rice fields. In the rice–duck co-culture system, ducks can not only control weed populations by feeding on them, but their movement, trampling, and stirring can also bury weeds in the soil, effectively controlling their growth. At the same time, the disturbance caused by the ducks in the water can increase turbidity, reduce light penetration, disturb the sediment, and inhibit weed germination and growth [26,27].

Based on the observation of duck behavior, this study proposes a biomimetic duck-bill chain vibration weeding device and conducts experimental research to investigate the structural and operational parameters of the device. The aim is to reduce damage to rice seedlings and improve the weed removal rate.

## 2. Evaluation of Weed Seedling Growth

After approximately one week of transplanting the seedlings, rice plants enter the vegetative growth stage and establish strong roots. Meanwhile, weeds scattered in the field begin to germinate and take root, making weeding increasingly challenging as they grow. To identify the best timing for rice weeding, a weed seed cultivation experiment was conducted. The germination times of two prevalent rice field weeds, barnyard grass and sedge, were recorded, and the relationship between the root growth of barnyard grass and time was analyzed.

### 2.1. Weed Seed Cultivation Experiment

To evaluate the effect of seeding days on the germination rates of barnyard grass and sedge seeds, a single-factor experiment was conducted. The germination time of weed seeds is an important characteristic of weed outbreak. During the experiment, 100 plump weed seeds were selected and evenly sown on the surface of 45 mm thick paddy soil in a container with dimensions of 170 mm in length, 115 mm in width, and 55 mm in height. The container was then placed in a germination chamber at a temperature of 26 °C with soil constantly moistened but without standing water. Three sets of repetitions were conducted for each experiment, and the number of germinated weeds was recorded daily to calculate the average germination rate.

**Figure 1:**
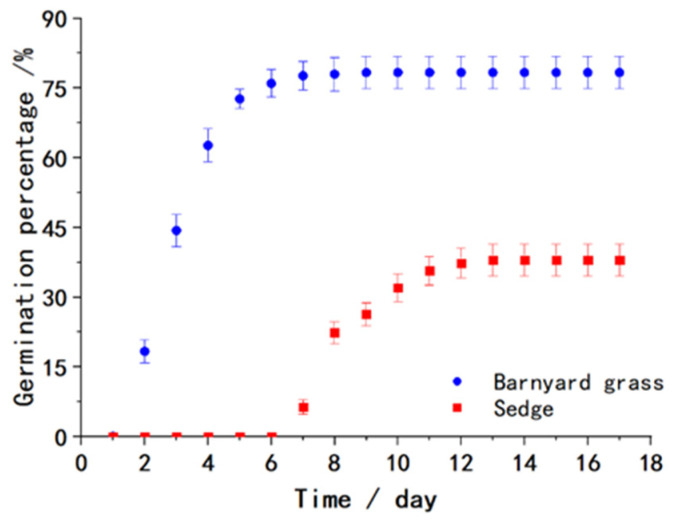
Germination rates of weed seeds from day 1 to day 17.

The test results are presented in Figure 1, where the germination rate data for the barnyard grass and sedge seeds from day 1 to day 17 are shown. The barnyard grass seeds germinated faster, with a maximum germination rate of 78.3% on the 7th day. The sedge seeds, however, had a slower germination rate and started to germinate on the 7th day, with a maximum germination rate of 38% on the 13th day. There was no evidence of germination for any of the ungerminated seeds until the end of the experiment. The experiment demonstrated that the germination times for these two weed seeds were concentrated within 1 to 12 days after sowing.

### 2.2. Observation of Barnyard Grass Root System

Root systems are not only the main absorption organs for plant growth nutrients but also crucial support for plants to resist disturbances. In order to observe the growth of weed roots after sowing, a study was conducted focusing on the growth of barnyard grass roots as our research subject. The research aimed to investigate the influence of sowing time on the length of barnyard grass roots.

A 45 mm thick layer of paddy field soil was evenly spread in a container with dimensions of 170 mm in length, 115 mm in width, and 55 mm in height. Thirty plump barnyard grass seeds were sown on the soil surface each day, and the sowing time was marked on the containers. The containers were then placed in a germination chamber at a temperature of 26 °C for 17 days, ensuring that the soil was moist but not waterlogged. After cultivation, the soil around the weed seedlings was cleaned with a brush and water. Ten weed seedlings with intact root systems were selected for each sowing day. The weed root systems were analyzed using a ScanMaker i800 plus scanner produced by Microtek and Wseen LA-S plant image analysis software(WSEEN 2017).

**Figure 2:**
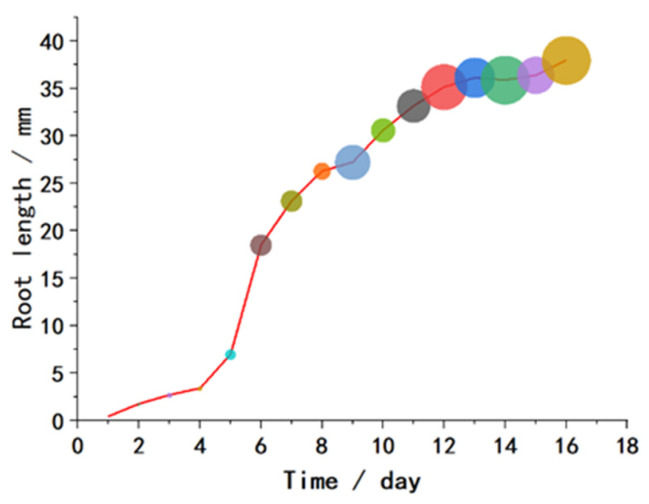
Total length of barnyard grass roots from day 1 to day 16 of cultivation.

**Figure 3:**
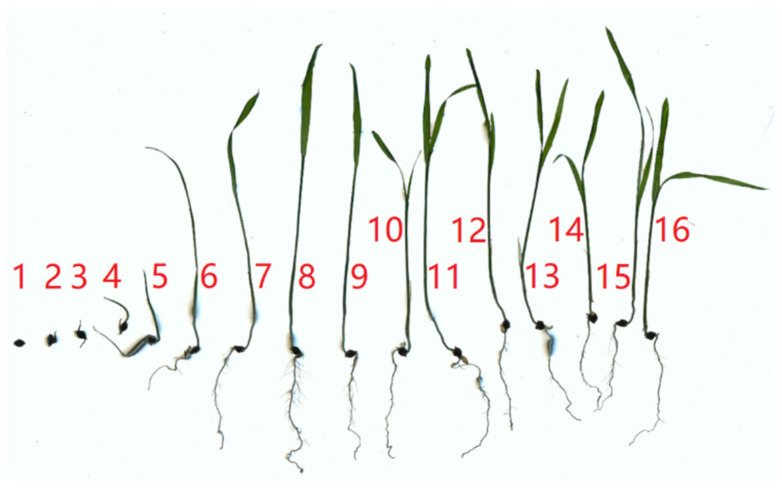
Barnyard grass seedlings grown for 1 to 16 days.

The relationship between the total length of the weed roots and the sowing time is shown in Figure 2. The growth of the barnyard grass roots is relatively slow in the first four days, followed by a rapid increase starting from the 5th day. The growth rate slows down around the 11th day and stabilizes at around 36 mm. The growth of the barnyard grass seedling roots from the 1st day to the 16th day is shown in Figure 3. Small branches start to emerge around the 7th day, resulting in rapid growth of the root system. However, the length growth rate of the main root slows down as the second leaf begins to emerge. The conclusion regarding root depth is consistent with the research by Wang et al. [28].

In summary, waterlogged weeds take about 12 days to complete germination after seeding, with the main root system growing rapidly to over 35 mm. Concurrently, transplanted rice seedlings complete the green-up, and the root system stabilizes at over 100 mm, entering the tillering stage. This is the optimal time for carrying out weeding operations.

## 3. Duckbill Weed Removal and Its Mathematical Model

### 3.1. High-Speed Photography Observation of Ducks Weeding

**Figure 4:**
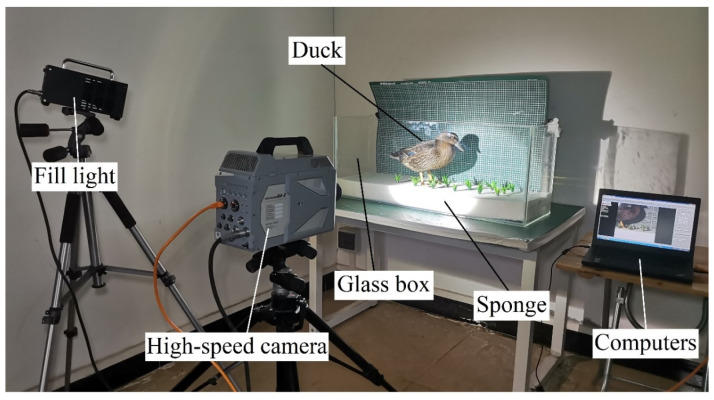
High-speed photography experiment for observing the living habits of ducks.

To clearly observe the disturbance process of ducks on weeds, Muscovy ducks raised in southern Chinese rice fields were selected as the research subjects. A glass tank measuring 1200 mm in length, 400 mm in width, and 500 mm in height was designed. The tank’s bottom was covered with a 130 mm thick sponge to simulate the soft soil of rice fields, and plastic fake grass with a height of 70 mm was fixed on the surface of the sponge. Figure 4 shows a FASTCAM SA-Z high-speed camera used to continuously observe the living habits of a 6-month-old Muscovy duck for two days under both no water and a 3 cm deep water layer on the surface of the sponge. The high-speed camera had a shooting frequency of 2400 frames per second.

**Figure 5:**
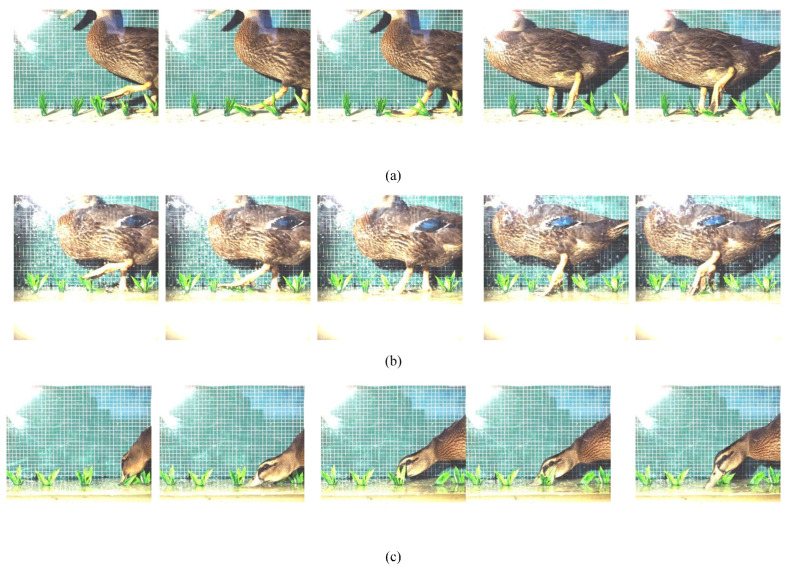
High-speed photography experiment to observe the living habits of ducks. (**a**) Disturbance of weeds by duck feet in the absence of water. (**b**) Disturbance of weeds by duck feet in a watered state. (**c**) Disturbance of weeds caused by duck beak vibration in the presence of water.

As observed in Figure 5a, it was found that, in the absence of water on the mud surface, the disturbance of the simulated grass mainly concentrated on the action of the duck’s webbed feet. When the duck’s foot was extended forward, the claw contracted. Once touching the ground, the duck’s webbed feet would quickly open into a fan shape, increasing the contact area between the feet and the mud surface. The contact area between the duck’s foot and the mud surface reached its maximum, effectively distributing the force generated by the duck over a larger soil area. After the other foot completed the stepping motion, the base of the duck’s foot left the ground first, followed by the toes. During this process, the weeds under the duck’s foot experienced limited pressure and were difficult to be pressed into the soil, resulting in a limited weeding effect. As shown in Figure 5b, it was observed that, in the presence of water, the motion of the duck’s foot is consistent with that in the absence of water. Therefore, it can be concluded that the disturbance caused by the duck’s foot to the weeds is not significant.

Compared with the situation of no water on the mud surface, when there was a shallow layer of water, in addition to the duck’s feet, the duck’s beak also contributed to the interference with the weeds, as shown in Figure 5c. It was found that ducks have a habit of pecking at water, and their beaks constantly disturb the area near the weed roots, while their heads sway left and right and move back and forth. This easily causes the surface layer of soil to flip over, allowing the weed roots to detach from the soil without affecting the rice seedlings. According to the research of Wang, during the tillering stage, the root system of rice is much larger and deeper in the soil than that of weeds [28].

In summary, the weeding effect of the duck’s beak may play an irreplaceable role in the weeding process. The swinging motion of the duckbill agitates the soft soil in the surface layer of the rice field, causing the weed roots to detach from the soil. Simultaneously, due to the presence of a certain depth of water in the field, the weeds float on the water surface, preventing their roots from anchoring in the soil and depriving them of the opportunity to survive. Since transplanted rice roots are inherently larger than weed roots, they have a longer lifespan in the soil after transplanting. Rice seedlings exhibit excellent flexibility, making them more resilient to external disturbances. Based on this, we chose the duck’s beak as the biomimetic object for the design of the weeding mechanism in this study.

### 3.2. Duck Beak Feature Model

Following the techniques used in related studies [29,30,31], the duck beak was photographed to obtain its side and top views. Adobe Illustrator software was used to trace the edge of the beak and extract its contour curves, which were then exported as .dwg format files. The control point coordinates of the contour curves were exported as .txt files using Autodesk CAD 2020. Finally, the morphological characteristics of the duck beak were extracted using the fitting tool in MATLAB 2016b software, and a mathematical model for the frontal and side views of the beak was established using segmented fitting.

**Figure 6:**
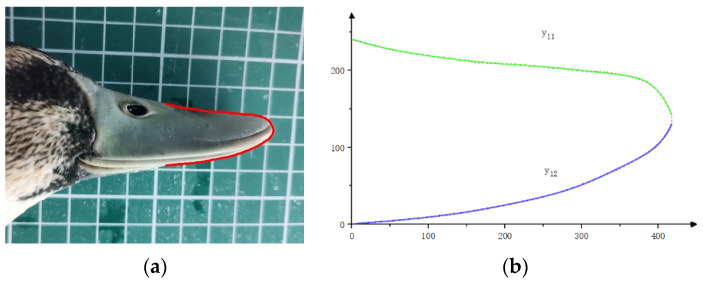
Feature extraction of the duck beak side view. (**a**) Side view of duck beak. (**b**) Duck beak side-view characteristic curve.

**Figure 7:**
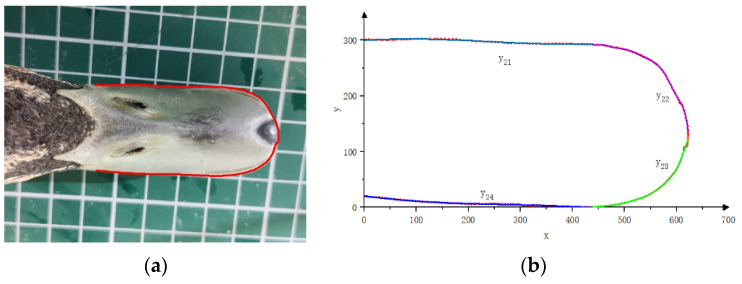
Feature extraction of duck beak top view. (**a**) Top view of a duck beak. (**b**) Duck beak top-view characteristic curve.

As shown in Figure 6 and Figure 7, the side view of the duck beak was divided into two curve sections and the top view into four curve sections according to their features. Curve equations were fitted for each section, and the goodness of fit was evaluated using the coefficient of determination R^2^. A value closer to 1 indicates a better fit, while a value closer to 0 indicates greater errors between the fitting curve and the sample data.

Based on the technology used in related research, the contour fitting equations of the duck bill side view are y_11_ and y_12_, respectively. The coefficients of the functions and their 95% confidence intervals are shown in Table 1. The residual sum of squares (SSE) and root mean square error (RMSE) of the upper contour fitting equation y_11_ are 48.67 and 0.8165, respectively, and the coefficient of determination (R^2^) is 0.9987. The SSE and RMSE of the lower contour fitting equation y_12_ are 41.66 and 0.8784, respectively, and the R^2^ is 0.9996.

The contour fitting equations of the duck bill top view are y_21_, y_22_, y_23_, and y_24_. The coefficients of the functions and their 95% confidence intervals are shown in Table 2. The SSE and RMSE of the contour fitting equation y_21_ are 47.06 and 0.8444, respectively, and the R^2^ is 0.9495. The SSE and RMSE of y_22_ are 147.27 and 1.752, respectively, and the R^2^ is 0.9989. The SSE and RMSE of y_23_ are 396.9 and 2.686, respectively, and the R^2^ is 0.9962. The SSE and RMSE of y_24_ are 26.40 and 0.5709, respectively, and the R^2^ is 0.9890. The R^2^ values of all fitting equations are above 0.98, indicating that the equations can accurately express the characteristics of the duckbill.

## 4. Design and Analysis of a Biomimetic Duckbill Chain-Type Weeding Device

### 4.1. Design of a Biomimetic Weeding Mechanism

**Figure 8:**
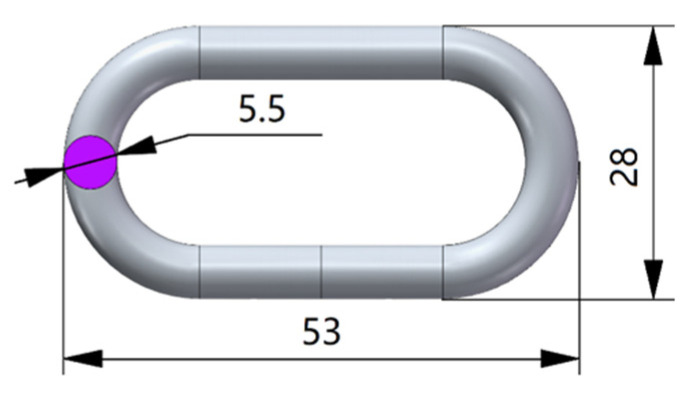
Chain Link Parameter Diagram.

The use of chains for weeding in rice fields has been reported [32], but there is a lack of design and research on specific parameters. The shape of the duck beak and the shape of a single chain link are similar. According to the characteristic curve of the duck beak, a chain with the specifications shown in Figure 8 was selected. The steel wire used to make the chain had a diameter of 5.5 mm, the total length of the chain link was 53 mm, the total width was 28 mm, and the material was galvanized manganese steel. The weight of a single chain link was 15.77 g.

**Figure 9:**
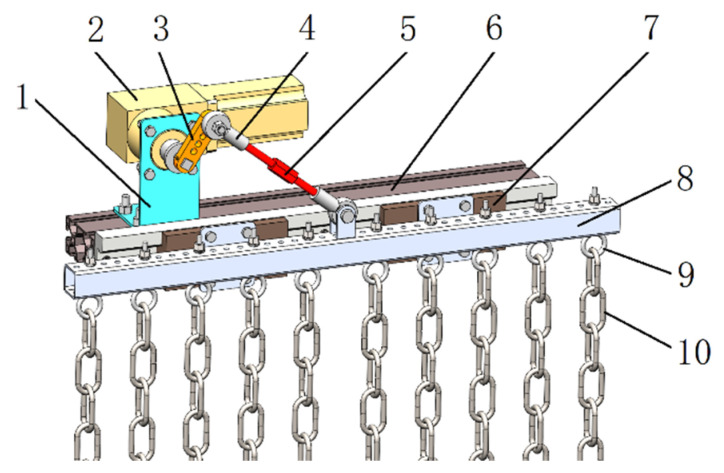
Experimental device of biomimetic duckbill weeding. 1. Motor mounting bracket. 2. Gearbox motor. 3. Crank arm. 4. Articulated bearing. 5. Connecting rod. 6. Frame. 7. Sliding module. 8. Crossbeam. 9. Chain mounting ring. 10. Chain.

In order to mimic the disturbance of soil and weeds in paddy fields by the duckbill, a biomimetic weeding device was designed as shown in Figure 9. It mainly consists of a biomimetic chain and a biomimetic vibration system. The biomimetic vibration system drives the biomimetic chain to swing, imitating the lateral movements of a duck’s head. One end of the biomimetic chain is free, while the other end is installed on a sliding module fixed on a crossbeam. The biomimetic vibration system mainly consists of a drive motor, a crankshaft, articulated bearings, and slide rails. The torque output from the motor is transmitted to the biomimetic weeding chain through the crank arm to achieve the swinging motion of the weeding chain. Adjusting the motor speed, the vibration frequency of the chain can be varied (driving frequency of chain forced vibration), while adjusting the installation hole of the joint bearing allows for amplitude adjustment. Changing the installation position of the chain on the crossbeam enables the adjustment of the chain density.

### 4.2. Dynamics Analysis of the Weed Removal Chain

**Figure 10:**
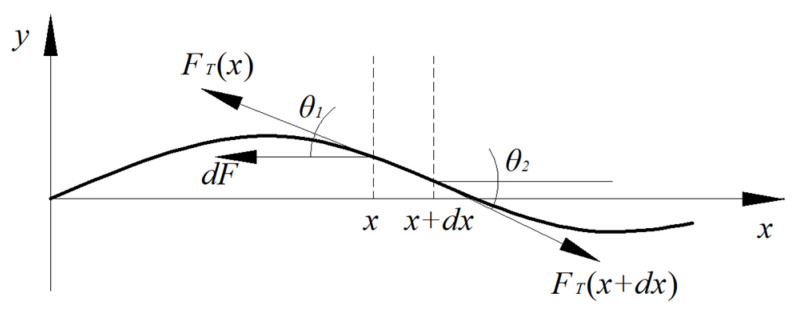
Force analysis of hanging chain element.

For a suspended chain, its dynamic equation is generally solved using Bessel functions and Neumann functions to obtain its natural frequency of vibration, energy distribution, chain tension, composite vibration, standing wave characteristic points, etc., [33,34,35]. As shown in Figure 10, for a chain with length *L* and density *ρ*, suspended along the *x*-axis, with endpoint A undergoing sinusoidal vibration in the y-direction, the vibration displacement of any point B on the chain at time *t* is *u*(*x*,*t*), and point C at a distance *dx* (*dx* approaching 0) from point B is subject to tension *FT*(*x*), *FT*(*x*+*dx*), and external force *dF*. The kinematic equilibrium equations in the *x* and *y* directions are shown in Equation (1).

(1) $$\left\{\begin{array}{c} F_{T}(X+dx)\cos\theta_{2}-F_{T}(x)\cos\theta_{1}-dF=0\\ F_{T}(X+dx)\sin\theta_{2}-F_{T}(x)\sin\theta_{1}=\rho \frac{\partial^{2}u}{\partial^{2}t}ds \end{array}\right.$$

Because *θ*_1_ ≈ *θ*_2_ ≈ 0, then cos *θ* ≈ 1, *θ* ≈ sin *θ* ≈ tan *θ* = *ux* = ∂u/∂x, *ds* ≈ *dx*. Therefore, there is *dF_T_* = *dF*, i.e., $\partial (F_{T}u_{x})/\partial x-\rho (\partial^{2}u/\partial^{2}t)=0$. Therefore, the vibration motion equation of the suspension chain is shown in Equation (2):

(2) $$F_{T}\frac{\partial^{2}u}{\partial^{2}x}+\frac{\partial F_{T}}{\partial x}\frac{\partial u}{\partial x}-\rho \frac{\partial^{2}u}{\partial^{2}t}=0$$

When the free end is stationary, *dF*_T_ = *dF* = *ρgdx* can be obtained from *dF_T_* = *dF*. Therefore, *F*_T_(*x*) = *ρgx*. Substituting this into Equation (2), we obtain the wave equation of the hanging chain as shown in Equation (3):

(3) $$x\frac{\partial^{2}u}{\partial^{2}x}+\frac{\partial u}{\partial x}-\frac{1}{g}\frac{\partial^{2}u}{\partial^{2}t}=0$$

Substituting u(x,t)=φ(x)f(t) into Equation (3) and separating variables yields Equation (4):

(4) $$\varphi^{-1}(x\varphi \prime \prime +\varphi \prime )={\left(fg\right)}^{-1}\overset{••}{f}=-k$$

Where *k* is a dimensionless constant. According to Equation (6), we can derive Equations (5) and (6) through manipulation:

(5) $$\overset{••}{f}+kgf=0$$

(6) xφ″+φ′+kφ=0

The solution of Equation (7) is

(7) f(t)=Acos(ωt)+Bsin(ωt)

where $\omega^{2}=kg$. Both *ω* and *k* are determined by boundary conditions. Substitute $W=2\sqrt{kx}$ into Equation (6) to obtain the 0-order Bessel equation as shown in Equation (8):

(8) $$\frac{d\varphi }{dw^{2}}+\frac{1}{w}\frac{d\varphi }{dw}+\varphi =0$$

Therefore, the solution of Equation (6) is

(9) $$\varphi (x)=J_{0}(w)=J_{0}(2\sqrt{kx})$$

Point *x* = 0 is a singular point, but it does not meet the physical conditions. Due to the boundary condition *u(l,t)* = 0, φ(l)=0, and, therefore, $J_{0}(2\sqrt{kl})=0$. So, *k* satisfies

(10) $$k_{i}=\frac{{\left[\mu_{i}^{\left(0\right)}\right]}^{2}}{4l}$$

where $\mu_{i}^{\left(0\right)}$ is the *i-th* positive zero of the Bessel equation of order 0. Therefore, the intrinsic vibration frequency of the suspension chain vibration is obtained as shown in Equation (11):

(11) $$\omega_{i}=\sqrt{k_{i}g}=\frac{\mu_{i}^{\left(0\right)}}{2}\sqrt{\frac{g}{l}}$$

The general solution to the vibration equation of the suspension chain is

(12) $$u(x,t)=\sum_{i=1}^{\infty }J_{0}(2\sqrt{k_{i}x})\left[A_{i}\cos(\omega_{i}t)+B_{i}\sin(\omega_{i}t)\right]$$

In the equation, *A_i_* and *B_i_* are determined by the initial conditions.

According to Equations (11) and (12), the vibration of the suspended chain is a superposition of multiple vibration modes, and the inherent vibration frequency and the length of the chains of each vibration mode are responsible for the vibration of the suspended chain.

In addition to vibration, the weeding chain also has a traction motion in the working direction under the drive of the machinery, which can be regarded as linear motion. Therefore, a forward traction motion needs to be superimposed on the vibration.

## 5. Experimental Study on Parameters and Operational Performance

### 5.1. Design of a Biomimetic Weeding Mechanism

The experiment was conducted in October 2022 at the Crop-Soil-Machine System Laboratory of South China Agricultural University, Guangdong Province, People’s Republic of China (23°10′04″ N, 113°21′50″ E). The experimental field was an agricultural machinery field trench with dimensions of 70 m × 16 m, forming a rectangular shape with an effective planting area of 1120 m^2^. A track-type agricultural machinery test trolley was installed along the edge of the field, with an adjustable working velocity of 0–5 km/h and controllable vertical displacement. The test trolley could carry various agricultural machineries, such as rotary tillers, transplanters, and pesticide sprayers, for operation. The experimental field was planted with two seasons of rice throughout the year and was left fallow after winter plowing. Before rice transplantation, water-assisted rotary tillage was conducted, and the experiment began on the 12th day after transplantation. The rice variety used in this experiment was Huahang 51, provided by the Seed Industry Company of South China Agricultural University, with three seedlings per hole and a row spacing of 30 cm. The main weeds in the field were common and malignant rice weeds in southern China, such as barnyard grass, sedge, and pygmy arrowhead. During the experiments, a water depth of 3–8 cm was maintained in the field. After the experiments, the water depth was maintained above 8 cm, ensuring that the rice seedling core was not submerged.

### 5.2. Experimental Design

#### 5.2.1. Experimental Factors

According to the above analysis, the factors that mainly affect the vibration effect of the chain include the length of the chains, vibration frequency, and working velocity. Obviously, the number of chains per row (the number of chains arranged in a single rice row with a width of 30 cm) and the vibration amplitude (the swing amplitude of the chain root) are also factors that need to be considered. Therefore, this paper focuses on five factors to investigate their effects on the weed removal performance of the biomimetic vibration chain weeding device.

After some preliminary experiments and an investigation of the working velocity of rice field machinery, it was determined that the speed of the paddy field machinery is generally between 0.5 and 1.2 m/s, the length of the chains is more reasonable at 0.6–1.4 m (with a gap of about 0.25 m above the mud surface), the vibration amplitude is more appropriate at 34–120 mm, and the number of chains per row of rice is more reasonable at 1–5. Referring to the vibration of the duckbill observed in the high-speed photography, the vibration frequency of the chain was set to 5–15 Hz.

Design-Expert software was used to design a central composite design text for the five aforementioned factors. The experiment was carried out using a 1/2 fraction type design, with 6 center point repeated tests and a total of 32 tests. Therefore, *r* = 2, and according to the coding formula shown in Equation (13), the natural space factor values were converted into coding space values, resulting in the test factor level settings shown in Table 3. *z*_1_, *z*_2_, *z*_3_, *z*_4_, *z*_5_, *x*_1_, *x*_2_, *x*_3_, *x*_4_, and *x*_5_ correspond to the natural factors *z_j_* and coding factors *x_j_* for the vibration amplitude, length of chains, number of chains per row, vibration frequency, and working velocity, respectively.

(13) $$x_{j}=\frac{2\times (z_{j}-z_{0j})}{z_{2j}-z_{1j}}$$

**Figure 11:**
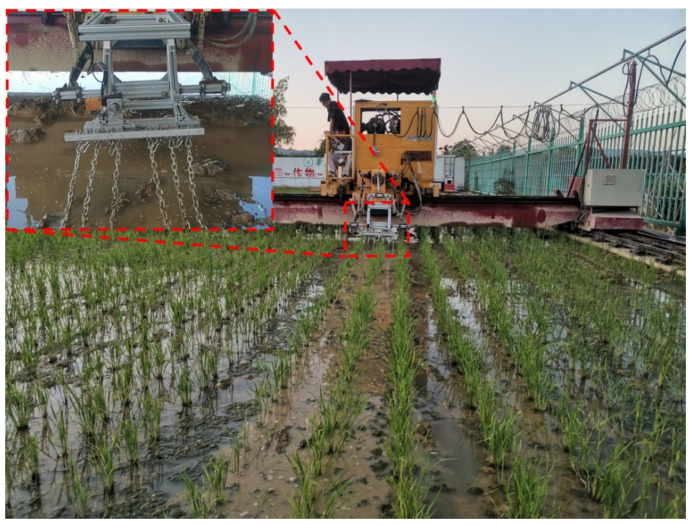
Testing site.Due to the inability of the image to clearly display the details of the equipment used in the experiment, red dottedt box have been added to provide more detailed description of the experimental equipment.

The experimental setup, as shown in Figure 11, utilized a motor-driven rocker mechanism to induce vibration in the chains. The field plot was divided into 36 experimental plots, with 32 plots randomly designated as experimental zones and subjected to the designated weeding treatments. The remaining four plots served as blank control test zones and received no treatment.

#### 5.2.2. Evaluation indices of weeding

In order to accurately evaluate the operational performance of the bio-inspired vibration chain weeding device, the weed removal rate and rice seedling damage are two important evaluation indicators for assessing the weeding machinery, according to relevant research methods [21]. The weed removal rate was statistically analyzed on the day of weeding and on the 7th day after weeding. In the area where weeding was not performed within the same field, five points were randomly selected, the number of weeds within a 1 m^2^ area was counted, and the average value *N* was obtained. Three points were selected within each test plot to count the number of weeds *M* within a 1 m^2^ area. The weed removal rate *y_j_* was calculated according to Equation (14):

(14) $$y_{j}=\frac{N-M}{N}\times 100\%$$

Due to the small number of injured seedlings, the rice seedlings that were bent or flattened did not lose their vital signs after several days of growth, and it became increasingly difficult to count them as time went on. Therefore, this measurement was only conducted on the day of the experiment. The number of injured seedlings was counted in each experimental plot, including both broken and flattened rice seedlings. The measurement of injured seedlings was only carried out on the day of the experiment and was not repeated seven days later.

### 5.3. Testing Results and Analysis

#### 5.3.1. Central Composite Text Plan Design

The experimental design and results are shown in Table 4, where *y_1_*, *y_2_*, and *y_3_* represent the first and second statistical results of the weed removal rate and the number of damaged seedlings.

#### 5.3.2. Regression Model Establishment and Significance Test

The test results were analyzed using Design-Expert 8.0 software, and the regression model variance analysis, regression coefficient significance test, and lack of fit test were performed on the first day weed removal rate (*y*_1_), the seventh day weed removal rate (*y*_2_), and the number of damaged seedlings (*y*_3_). Table 5 presents the results of the analysis.

An analysis of Table 5 shows that the quadratic regression model established between the experimental factors and the two weed removal rates obtained from the two measurements is extremely significant, with a significance level of 0.01 (*p*-values of 0.0002 and 0.004), indicating a good fit of the regression model. The lack of fit test result is not significant (*p* > 0.25, with *p*-values of 0.7738 and 0.6999), indicating a high degree of fit of the regression equation. The quadratic regression model established between the experimental factors and the number of damaged seedlings is also extremely significant, with a significance level of 0.05 (*p* = 0.0155); the lack of fit test result is not significant (*p* = 0.4481 > 0.25), indicating a high degree of fit of the regression equation concerning the number of damaged seedlings.

According to the significance test results of the regression equation coefficients in Table 5, eight factors have an extremely significant impact on the weed removal rate on the first day after weeding, namely, *x*_3_, *x*_4_, *x*_5_, *x*_1_*x*_5_, *x*_3_*x*_4_, *x*_4_*x*_5_, *x*_3_^2^, and *x*_4_^2^, with two factors having a significant impact, namely, *x*_1_ and *x*_2_. Five factors have an extremely significant impact on the weed removal rate on the seventh day after weeding, namely, *x*_3_, *x*_4_, *x*_5_, *x*_4_*x*_5_, and *x*_3_^2^, with five factors having a significant impact, namely, *x*_1_, *x*_1_*x*_5_, *x*_3_*x*_4_, *x*_3_*x*_5_, and *x*_4_^2^. Two factors have an extremely significant impact on the number of damaged rice seedlings, namely, *x*_1_*x*_5_ and *x*_3_^2^, with three factors having a significant impact, namely, *x*_1_, *x*_3_, and *x*_5_. The determination coefficients R^2^ of the three models are 0.9496, 0.9389, and 0.8696, respectively, indicating that the three fitting models have sufficient explanatory power for the evaluation indicators.

To optimize the regression model, insignificant coefficient terms were removed, and the *y*_1_, *y*_2_, and *y*_3_ quadratic regression equations were converted to the natural space according to coding Equation (13), as shown in Equations (15)–(17).

**Figure 12:**
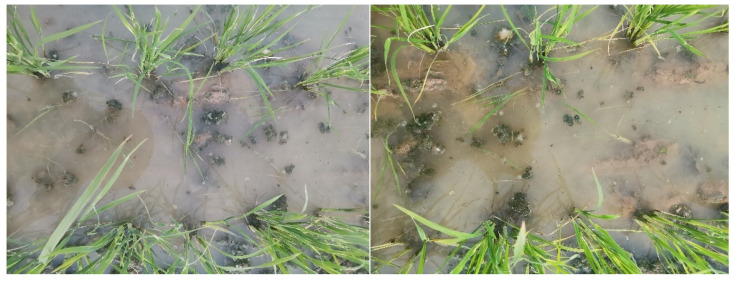
Weeds one day after the test.

**Figure 13:**
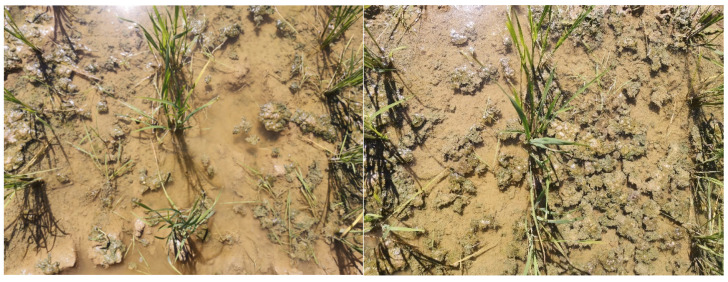
Weeds after 7 days of test.

From the F-values and absolute values of the regression equation coefficients in Table 5, the impact sequence of each experimental factor on the weed removal rate on the first and seventh days after weeding is consistent, and the impact sequence is as follows: working velocity > number of chains per row > vibration frequency > vibration amplitude > length of chains. The impact sequence of each experimental factor on the number of damaged rice seedlings after weeding is as follows: working velocity > number of chains per row > vibration amplitude > vibration frequency > length of chains. Figure 12 and Figure 13 respectively depict the weed status in the field on 1 day after weeding and 7 days after weeding.

(15) $$\hat{y_{1}}=170.42-1.35z_{1}-0.21z_{2}+106.55z_{3}+5.81z_{4}-577.77z_{5}+1.88z_{1}z_{5}-2.88z_{3}z_{4}+30.53z_{4}z_{5}-9.82z_{3}^{2}-0.79z_{4}^{2}$$

(16) $$\hat{y_{2}}=297.83-1.08z_{1}-0.15z_{2}+64.75z_{3}+0.54z_{4}-674.03z_{5}+1.51z_{1}z_{5}-2.48z_{3}z_{4}+33.64z_{3}z_{5}+31.59z_{4}z_{5}-8.58z_{3}^{2}-0.68z_{4}^{2}$$

(17) $$\hat{y_{3}}=-4.78+0.09z_{1}-1.94z_{3}-0.02z_{4}+10.14_{5}-0.12z_{1}z_{5}+0.36z_{3}^{2}$$

#### 5.3.3. Influence of Interaction Factors on Performance Indicators

**Figure 14:**
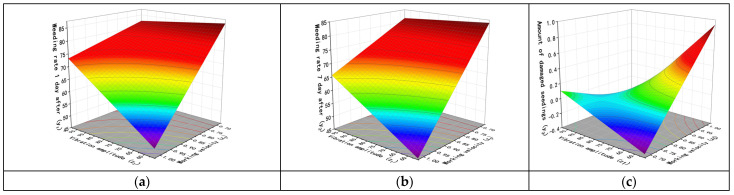
Influence of interaction factors between vibration amplitude and working velocity. (**a**) Weeding rate 1 day after. (**b**) Weeding rate 7 days after. (**c**) Number of damaged seedlings.

**Figure 15:**
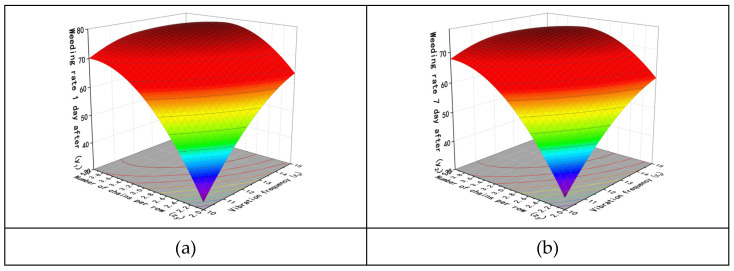
Influence of interaction factors between number of chains and vibration frequency. (**a**) Weeding rate 1 day after. (**b**) Weeding rate 7 day after.

**Figure 16:**
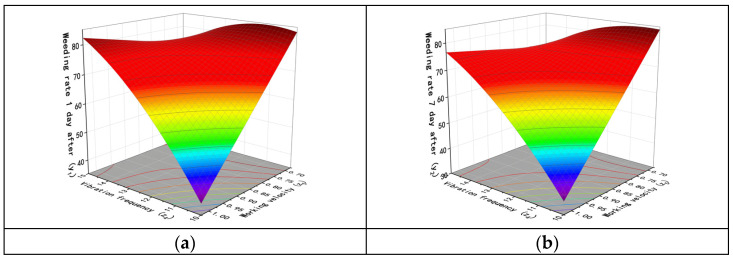
Influence of interaction factors between vibration frequency and working velocity. (**a**) Weeding rate 1 day after. (**b**) Weeding rate 7 day after.

**Figure 17:**
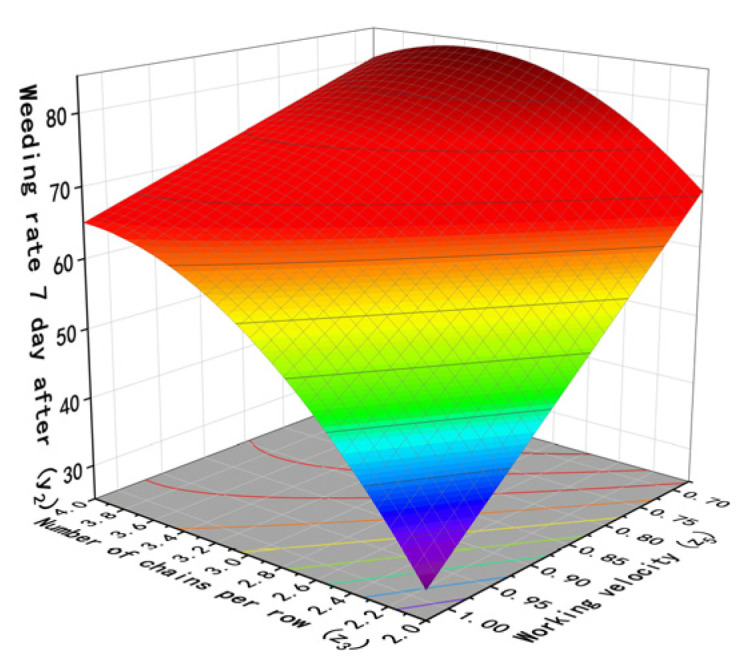
Influence of interaction factors on weeding rate 7 day after between number of chains and working velocity.

According to the above analysis, the interactions between the factors have varying degrees of impact on the test results. The interaction between the vibration amplitude and working velocity (*x*_1_*x*_5_) has a significant effect on the weed removal rate and seedling damage on the first and seventh days after weed removal. The interaction between the number of chains per row and vibration frequency (*x*_3_*x*_4_) has a significant effect on the weed removal rate on the first and seventh days after weed removal. The interaction between the number of chains per row and working velocity (*x*_3_*x*_5_) has a significant effect on the weed removal rate on the seventh day after weed removal. Additionally, the interaction between the chain vibration frequency and working velocity (*x*_4_*x*_5_) has a significant effect on the weed removal rate on the first and seventh days after weed removal. The response surface of the interaction was obtained using Design-Expert software, as shown in Figure 14, Figure 15, Figure 16 and Figure 17.

When the length of chains *x*_2_ = 100cm and the number of chains per row *x*_3_ = 3, and the vibration frequency *x*_4_ = 12Hz, the interaction between the vibration amplitude *x*_1_ and working velocity *x*_5_ is shown in Figure 14a for the weed removal rate one day after weeding. As the working velocity increases, the weed removal rate decreases. At low working speeds, a lower vibration amplitude achieves a higher weed removal rate than a higher vibration amplitude. However, at high working speeds, a higher vibration amplitude achieves a better weed removal effect than a lower vibration amplitude. Figure 14b shows the test results seven days after weeding, with a similar trend to Figure 14a, but with a slight decrease in the weed removal rate compared to the first day’s test results. The interaction between these two factors’ impact on seedling damage is shown in Figure 14c, where a higher vibration amplitude results in less seedling damage at higher working speeds, but the opposite is observed at low working speeds, where a greater vibration amplitude results in more seedling damage than a smaller vibration amplitude.

Figure 15 examines the interaction between the number of chains per row *x*_3_ and vibration frequency *x*_4_, where the vibration amplitude *x*_1_ = 77 mm, the length of chains *x*_2_ = 100 cm, and working velocity *x*_5_ = 0.85 m/s. Figure 15a shows the interaction between these two factors and the weed removal rate on the first day. When both the number of chains per row and the vibration frequency are small, the weed removal rate is low. However, increasing the vibration frequency and the number of chains can effectively improve the weed removal rate. The trend shown in Figure 15b is consistent with that in Figure 15a.

Figure 16 shows the interaction between the vibration frequency *x*_4_ and working velocity *x*_5_ on the weed removal rate on the first and seventh days of weeding. Similar trends were observed in both periods. When the vibration amplitude *x*_1_ = 77 mm, the length of chains *x*_2_ = 100 cm, and the number of chains per row *x*_3_ = 3, better results were obtained at relatively low levels of vibration frequency and working speed. When a low vibration frequency and a high working speed were used, the weed removal rate was only about 40%. Similarly, excellent results were obtained with a higher working speed and vibration frequency. However, when both the working speed and vibration frequency were at high levels, the weed removal rate decreased.

When the chain vibration amplitude (*x*_1_) is 77 mm, the length of chains (*x*_2_) is 100 cm, and the vibration frequency (*x*_4_) is 12.5 Hz, the effect of the number of chains per paddy row (*x*_3_) and working velocity on the weed removal rate on the 7th day is shown in Figure 17. When the number of chains per paddy row is constant, the weed removal rate increases as the working velocity decreases. However, at low speeds, the weed removal rate first increases as the number of chains increases and reaches a maximum value at around 3.5, and then it slowly decreases.

## 6. Parameter Optimization and Test

### 6.1. Parameter Optimization

Using the optimization function in Design-Expert software, the biomimetic vibrating weeding device was optimized based on Equations (18)–(20). Combining the structural parameter range specified in Equation (18), the operating parameter range defined in Equation (19), and the optimization objectives and boundary conditions limited by Equation (20), a total of 54 optimal parameter combinations were obtained. Under these parameter combinations, weed removal rates exceeding 90% and minimal seedling damage were achieved. However, it should be noted that, among the five experimental factors, working velocity and vibration frequency are working parameters, especially working velocity, which is variable during actual operation, while the structural parameters need to be determined during operation.

Regarding the optimization of structural and operational parameters for the bionic chain-type weeding machine, when only considering the objectives specified in Equation (20), numerous combinations of *x* can be obtained, all of which achieve a weed removal rate above 90% and minimal crop damage. However, *x*_4_ and *x*_5_ are operational parameters (O.P) that need to vary within a certain range during actual operation, especially working velocity *x_5_*. However, *x*_1_, *x*_2_, and *x*_3_ are structural parameters (S.P) that need to be determined as a specific value.

(18) $$s.p\left\{\begin{array}{l} 55.5<x_{1}<98.5\\ 60<x_{2}<100\\ 2<x_{3}<4 \end{array}\right.$$

(19) $$o.p\left\{\begin{array}{l} 7.5<x_{4}<17.5\\ 0.5<x_{5}<1.2 \end{array}\right.$$

(20) $$aim\left\{\begin{array}{l} y_{1}=f_{1}(x_{1},x_{2},x_{3},x_{4},x_{5})\\ y_{2}=f_{2}(x_{1},x_{2},x_{3},x_{4},x_{5})\\ y_{3}=f_{3}(x_{1},x_{2},x_{3},x_{4},x_{5})\\ \max y_{1}\\ \max y_{2}\\ \min y_{3} \end{array}\right.$$

In order to determine the three structural parameters *x*_1_, *x*_2_, and *x*_3_, the distribution of each parameter was statistically analyzed. The data from the 54 groups showed that the central values of *x*_1_, *x*_2_, and *x*_3_ followed normal distributions of N (78.8, 175.6), N (93.47, 423.1), and N (3.42, 0.16), respectively.

(21) $$s.p\left\{\begin{array}{l} x_{1}=78.8\\ x_{2}=93.47\\ x_{3}=3.42 \end{array}\right.$$

**Figure 18:**
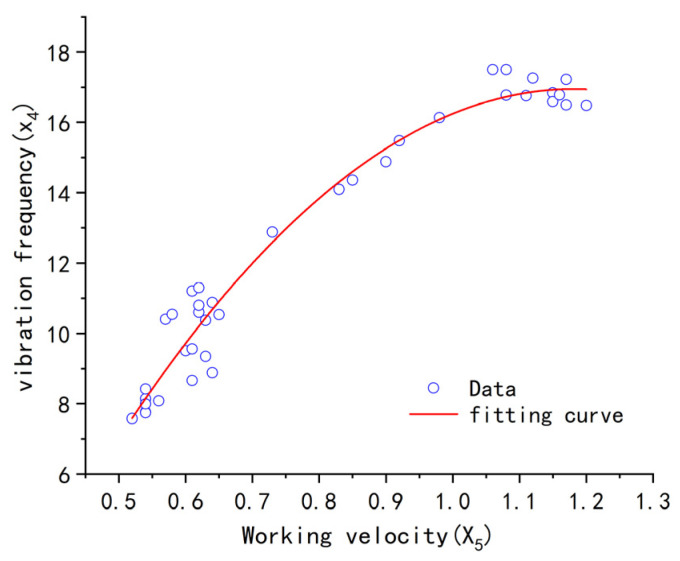
Fitting Diagram of the Relationship between Working velocity and Vibration Frequency.

Setting the structural parameters as shown in Equation (21), the optimization conditions were established by combining Equations (19)–(21), resulting in 38 sets of optimal working parameter combinations. Among them, both the weed removal rate on the first day and the weed removal rate on the seventh day were greater than 85%, and the seedling damage rate was close to 0. The distributions of *x*_3_ and *x*_4_ for the 38 parameter combinations obtained are shown in Figure 18. Using quadratic polynomial data fitting, the relationship curve between the working velocity and vibration frequency under the optimal operating result is shown in Equation (22), with a residual sum of squares of the fitting equation SSD = 16.89, R^2^ = 0.9648, and adj-R^2^ = 0.96279. This indicates that, during actual operation, the vibration frequency can be adjusted based on the working velocity to match the two parameters and achieve the optimal operation effect.

(22) $$x_{4}=-21.4x_{5}^{2}+50.55x_{5}-12.91$$

### 6.2. Test

According to the regression experiments and optimization results, a vibrational chain weeding device with a vibration amplitude of 80 mm, a chain length of 95 cm, and a single-row installation density of 3.5 (with an installation spacing of 8.5 mm) was installed behind the rice fertilizer applicator. The experiment was conducted with the power chassis of the Yangma rice transplanter, and the chain vibration was powered by the PTO (Power Take-Off) unit integrated into the chassis.

**Figure 19:**
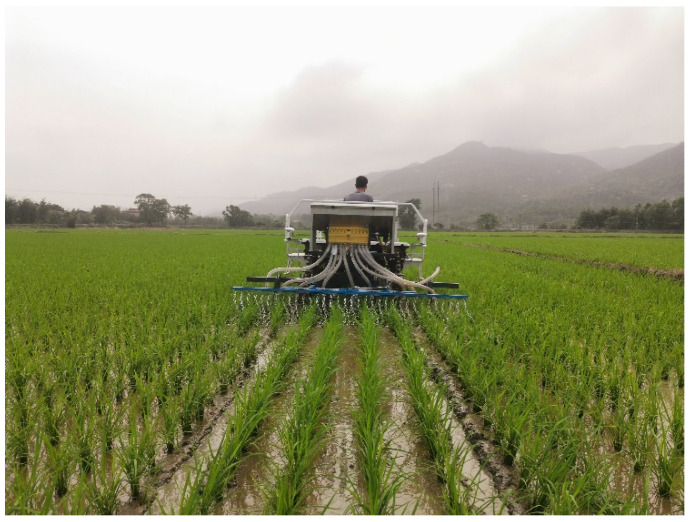
Testing site.

**Figure 20:**
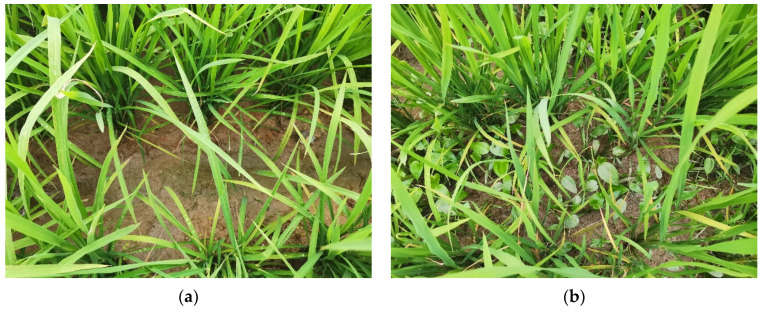
Weed growth status 1 month after. (**a**) The test group. (**b**) The control group.

The test was conducted on April 16, 2023, in Guanghai Town, Taishan City, Guangdong Province, People’s Republic of China (21°57′50.31″ N, 112°46′42.54″ E). The test field took the form of a rectangular plot measuring 162 m in length and 42 m in width, with a total area of 0.68 hectares (ha). Among this area, 0.437 ha was allocated for the application of the vibrating chain weeding treatment, while the remaining area served as the control group. Throughout the experiment, uniform field management practices were applied to all plots. Weed growth status was assessed on 2 May (15 days after test) and 16 May (30 days after test) on a per-unit area basis, with three repetitions. Shortly afterwards, the rice was closed, and no further evaluation was conducted. The testing site as shown in Figure 19. Weed growth status 1 month after is depicted in Figure 20. 

On 2 May, the weed density in the experimental group was 85.46% ± 4.73 lower per square meter than in the control group. On 16 May, the weed density in the experimental group was 79.51% ± 6.35 lower per square meter than in the control group. These results indicate that the investigated weed control method has a positive effect on rice production.

## 7. Conclusions

In this study, we developed a chain-type weeding device based on the biomimetic design of a duck bill for weed management in paddy fields during the tillering stage. The weed removal rate and seedling damage were used as evaluation indicators to study the effect of the device’s structural and operational parameters. The main research results are as follows:

The tillering stage of rice growth, when weeds have shallow roots and seeds have completed germination, is the best time for physical weeding.

Based on the playful water habits of duck bills in shallow water, a vibration chain-type weeding device for paddy fields was designed.

A central composite regression experiment was conducted to study the effects of three structural parameters and two operational parameters on the weed removal rate and seedling damage. The matching function between the device’s structural and operational parameters was determined.

This study provides an in-depth analysis of the weeding principle of chain-type weeding devices and investigates the interaction between the device’s structural and operational parameters. This technology offers a novel solution for weed control in rice cultivation. For future research, it is essential to investigate the production efficiency and economic benefits of weed control devices through experimental studies. This will provide further insights into the additional advantages of using mechanical weed control in the rice cultivation environment.

## Figures and Tables

**Table 1:** The coefficient of contour fitting function of duckbill beak side view.

Function	Coefficient	Value	95% Confidence Bounds
y_11_	b_10_	240.9	(239.4, 242.5)
	b_11_	−0.3296	(−0.5538, −0.1055)
	b_12_	1.654 × 10^−3^	(8.533 × 10^−3^, 1.184 × 10^−2^)
	b_13_	−7.152 × 10^−6^	(2.176 × 10^−4^, 2.033 × 10^−4^)
	b_14_	5.408 × 10^−8^	(−2.286 × 10^−6^, 2.394 × 10^−6^)
	b_15_	−7.647 × 10^−10^	(−1.595 × 10^−8^, −1.442 × 10^−8^)
	b_16_	6.235 × 10^−12^	(−5.298 × 10^−11^, 6.545 × 10^−11^)
	b_17_	−2.535 × 10^−14^	(−1.62 × 10^−13^, 1.113 × 10^−13^)
	b_18_	4.983 × 10^−17^	(−1.219 × 10^−16^, 2.216 × 10^−16^)
	b_19_	−3.795 × 10^−20^	(−1.286 × 10^−19^, 5.266 × 10^−20^)
y_12_	b_20_	−0.3475	(−2.457, 1.762)
	b_21_	0.2094	(−0.1077, 0.5264)
	b_22_	−6.737 × 10^−3^	(−2.106 × 10^−2^, 7.585 × 10^−3^)
	b_23_	1.591 × 10^−5^	(−1.312 × 10^−4^, −4.495 × 10^−4^)
	b_24_	−2.012 × 10^−6^	(−5.176 × 10^−6^, 1.152 × 10^−6^)
	b_25_	1.502 × 10^−8^	(−5.133 × 10^−9^, 3.517 × 10^−8^)
	b_26_	−6.723 × 10^−11^	(−1.445 × 10^−10^, 1.009 × 10^−11^)
	b_27_	1.767 × 10^−13^	(8.537 × 10^−16^, 3.525 × 10^−13^)
	b_28_	−2.506 × 10^−16^	(−4.688 × 10^−16^, −3.239 × 10^−17^)
	b_29_	1.477 × 10^−19^	(3.395 × 10^−20^, 2.615 × 10^−19^)

**Table 2:** The coefficient of contour fitting function of duckbill top side view.

Function	Coefficient	Value	95% Confidence Bounds
y_21_	d_10_	299.7	(299, 300.3)
	d_11_	0.06227	(0.04834, 0.07619)
	d_12_	−4.45 × 10^−4^	(−5.216 × 10^−4^, 3.684 × 10^−4^)
	d_13_	6.147 × 10^−7^	(4.988 × 10^−7^, 7.305 × 10^−7^)
y_22_	d_20_	2.737 × 10^9^	(1.426 × 10^9^, 4.048 × 10^9^)
	d_21_	−4.762 × 10^7^	(−7.009 × 10^7^, −2.516 × 10^7^)
	d_22_	3.676 × 10^5^	(1.968 × 10^5^, 5.384 × 10^5^)
	d_23_	−1652	(−2409, −896.1)
	d_24_	4.766	(2.617, 6.915)
	d_25_	−9.148 × 10^−3^	(−1.321 × 10^−2^, −5.085 × 10^−3^)
	d_26_	1.168 × 10^−5^	(6.572 × 10^−6^, 1.68 × 10^−5^)
	d_27_	−9.577 × 10^−9^	(−1.371 × 10^−8^, −5.449 × 10^−9^)
	d_28_	4.571 × 10^−12^	(2.629 × 10^−12^, 6.512 × 10^−12^)
	d_29_	−9.678 × 10^−16^	(−1.373 × 10^−15^, −5.627 × 10^−16^)
y_23_	d_30_	9.546 × 10^8^	(−8.684 × 10^8^, 2.778 × 10^9^)
	d_31_	−1.655 × 10^7^	(−4.781 × 10^7^, −1.471 × 10^7^)
	d_32_	1.273 × 10^5^	(−1.105 × 10^5^, 3.651 × 10^5^)
	d_33_	−570.1	(−1624, 483.5)
	d_34_	1.638	(−1.357, 4.633)
	d_35_	−3.132 × 10^−3^	(−8.798 × 10^−3^, −2.533 × 10^−3^)
	d_36_	3.986 × 10^−6^	(−3.147 × 10^−6^, 1.112 × 10^−5^)
	d_37_	−3.254 × 10^−9^	(−9.017 × 10^−9^, −2.508 × 10^−9^)
	d_38_	1.547 × 10^−12^	(−1.164 × 10^−12^, 4.258 × 10^−12^)
	d_39_	−3.263 × 10^−16^	(−8.92 × 10^−16^, 2.395 × 10^−16^)
y_24_	d_40_	20.45	(20, 20.9)
	d_41_	−0.1277	(−0.1368, −0.1185)
	d_42_	3.841 × 10^−4^	(3.352 × 10^−4^, 4.329 × 10^−4^)
	d_43_	−4.62 × 10^−7^	(−5.355 × 10^−7^, −3.884 × 10^−7^)

**Table 3:** Test factor level.

*x_j_* (*z_j_*)	Vibration Amplitude (*z*_1_)/mm	Length of Chains (*z*_2_)/mm	Number of Chains Per Row (*z*_3_)	Vibration Frequency (*z*_4_)/Hz	Working Velocity (*z*_5_)/m·s^−1^
*r*(*z*′_2*j*_)	120	140	5	7.5	1.2
1(*z*_2*j*_)	98.5	120	4	10	1.025
0(*z*_0_)	77	100	3	12.5	0.85
−1(*z*_1*j*_)	55.5	80	2	15	0.675
−*r*(*z*′_1*j*_)	34	60	1	17.5	0.5

**Table 4:** Test plan and results.

No	*x*_1_	*x*_2_	*x*_3_	*x*_4_	*x*_5_	*y*_1_	*y*_2_	*y*_3_
1	1	1	1	1	−1	50.7614	54.7718	1
2	1	1	1	−1	1	56.3452	49.3776	0
3	1	1	−1	1	1	75.1269	68.4647	0
4	1	1	−1	−1	−1	56.3452	61.8257	0
5	1	−1	1	1	1	91.3706	85.0622	0
6	1	−1	−1	−1	1	20.8122	19.917	0
7	1	−1	1	−1	−1	88.3249	84.2324	0
8	1	−1	−1	1	−1	70.0508	65.1452	0
9	−1	−1	−1	−1	−1	67.0051	68.8797	0
10	−1	−1	−1	1	1	51.269	51.8672	1
11	−1	−1	1	−1	1	39.5939	39.0041	3
12	−1	−1	1	1	−1	69.0355	62.2407	0
13	−1	1	−1	−1	1	−10.1523	−15.7676	1
14	−1	1	−1	1	−1	60.9137	64.3154	0
15	−1	1	1	−1	−1	80.203	76.7635	0
16	−1	1	1	1	1	61.4213	63.0705	1
17	−2(−r)	0	0	0	0	68.0203	67.6349	0
18	2(r)	0	0	0	0	86.2944	81.7427	0
19	0	2(r)	0	0	0	63.9594	65.5602	0
20	0	−2(−r)	0	0	0	80.203	75.9336	0
21	0	0	2(r)	0	0	71.5736	72.6141	2
22	0	0	−2(−r)	0	0	5.07614	7.05394	1
23	0	0	0	2(r)	0	75.6345	67.2199	0
24	0	0	0	−2(−r)	0	40.1015	46.888	0
25	0	0	0	0	−2(−r)	80.7107	82.1577	0
26	0	0	0	0	2(r)	51.269	40.6639	0
27	0	0	0	0	0	91.8782	87.9668	0
28	0	0	0	0	0	86.2944	81.3278	0
29	0	0	0	0	0	70.5584	65.5602	0
30	0	0	0	0	0	84.264	80.9129	1
31	0	0	0	0	0	63.9594	59.751	0
32	0	0	0	0	0	78.1726	73.444	0

**Table 5:** Analysis of variance of regression equation.

Evaluation Indicator	Source Variation	Sum of Squares	Degree Freedom	Mean Square	F Value	*p* Value
Weeding rate 1st day *y*_1_/%	Model	16,709.07	20	835.45	10.37	0.0002 **
	*x*_1_	665.66	1	665.66	8.26	0.0151 *
	*x*_2_	408.25	1	408.25	5.07	0.0458 *
	*x*_3_	3235.94	1	3235.94	40.16	<0.0001 **
	*x*_4_	1709.24	1	1709.24	21.21	0.0008 **
	*x*_5_	1939.25	1	1939.25	24.07	0.0005 **
	*x*_1_ *x*_2_	0.4	1	0.4	5 × 10^−3^	0.9449
	*x*_1_ *x*_3_	17.54	1	17.54	0.22	0.6499
	*x*_1_ *x*_4_	0.016	1	0.016	2 × 10^−4^	0.989
	*x*_1_ *x*_5_	800.86	1	800.86	9.94	0.0092 **
	*x*_2_ *x*_3_	10.07	1	10.07	0.12	0.7304
	*x*_2_ *x*_4_	0.016	1	0.016	2 × 10^−4^	0.989
	*x*_2_ *x*_5_	41.89	1	41.89	0.52	0.4859
	*x*_3_ *x*_4_	829.85	1	829.85	10.3	0.0083 **
	*x*_3_ *x*_5_	376.99	1	376.99	4.68	0.0534
	*x*_4_ *x*_5_	2854.38	1	2854.38	35.42	<0.0001 **
	*x*_1_^2^	7.73	1	7.73	0.096	0.7625
	*x*_2_^2^	93.19	1	93.19	1.16	0.3052
	*x*_3_^2^	3064.72	1	3064.72	38.04	<0.0001 **
	*x*_4_^2^	835.12	1	835.12	10.36	0.0082 **
	*x*_5_^2^	320.46	1	320.46	3.98	0.0715
	Residual error	886.34	11	80.58		
	Lack of fit	341.62	6	56.94	0.52	0.7738
	Pure error	544.72	5	108.94		
	Cor total	17,595.4	31			
Weeding rate 7th day *y*_2_/%	Model	15,414.69	20	770.7344	8.451414	0.0004 **
	*x*_1_	473.8282	1	473.8282	5.195718	0.0436 *
	*x*_2_	229.8586	1	229.8586	2.520492	0.1407
	*x*_3_	2838.285	1	2838.285	31.12294	0.0002 **
	*x*_4_	1223.643	1	1223.643	13.41774	0.0037 **
	*x*_5_	2820.264	1	2820.264	30.92534	0.0002 **
	*x*_1_ *x*_2_	11.71855	1	11.71855	0.128499	0.7268
	*x*_1_ *x*_3_	11.71855	1	11.71855	0.128499	0.7268
	*x*_1_ *x*_4_	13.18202	1	13.18202	0.144546	0.7110
	*x*_1_ *x*_5_	516.1004	1	516.1004	5.659249	0.0366 *
	*x*_2_ *x*_3_	0.010761	1	0.010761	0.000118	0.9915
	*x*_2_ *x*_4_	42.70975	1	42.70975	0.46833	0.5079
	*x*_2_ *x*_5_	3.884661	1	3.884661	0.042597	0.8403
	*x*_3_ *x*_4_	614.6696	1	614.6696	6.740101	0.0249 *
	*x*_3_ *x*_5_	554.495	1	554.495	6.080262	0.0314 *
	*x*_4_ *x*_5_	3057.035	1	3057.035	33.52162	0.0001 **
	*x*_1_^2^	1.440647	1	1.440647	0.015797	0.9022
	*x*_2_^2^	42.74073	1	42.74073	0.468669	0.5078
	*x*_3_^2^	2341.965	1	2341.965	25.6806	0.0004 **
	*x*_4_^2^	628.9049	1	628.9049	6.896197	0.0236 *
	*x*_5_^2^	367.8255	1	367.8255	4.033356	0.0698
	Residual error	1003.155	11	91.1959		
	Lack of fit	436.1307	6	72.68846	0.640964	0.6999
	Pure error	567.0242	5	113.4048		
	Cor total	16,417.84	31			
Number of damaged seedings *y*_3_	Model	13.23	20	0.66	3.67	0.0155 *
	*x*_1_	1.04	1	1.04	5.77	0.0351 *
	*x*_2_	0.042	1	0.042	0.23	0.6403
	*x*_3_	1.04	1	1.04	5.77	0.0351 *
	*x*_4_	0.042	1	0.042	0.23	0.6403
	*x*_5_	1.04	1	1.04	5.77	0.0351 *
	*x*_1_ *x*_2_	0.56	1	0.56	3.12	0.1052
	*x*_1_ *x*_3_	0.063	1	0.063	0.35	0.5681
	*x*_1_ *x*_4_	0.56	1	0.56	3.12	0.1052
	*x*_1_ *x*_5_	3.06	1	3.06	16.97	0.0017 **
	*x*_2_ *x*_3_	0.062	1	0.062	0.35	0.5681
	*x*_2_ *x*_4_	0.56	1	0.56	3.12	0.1052
	*x*_2_ *x*_5_	0.56	1	0.56	3.12	0.1052
	*x*_3_ *x*_4_	0.063	1	0.063	0.35	0.5681
	*x*_3_ *x*_5_	0.062	1	0.062	0.35	0.5681
	*x*_4_ *x*_5_	0.56	1	0.56	3.12	0.1052
	*x*_1_^2^	0.015	1	0.015	0.084	0.7774
	*x*_2_^2^	0.015	1	0.015	0.084	0.7774
	*x*_3_^2^	3.64	1	3.64	20.17	0.0009 **
	*x*_4_^2^	0.015	1	0.015	0.084	0.7774
	*x*_5_^2^	0.015	1	0.015	0.084	0.7774
	Residual error	1.98	11	0.18		
	Lack of fit	1.15	6	0.19	1.15	0.4481
	Pure error	0.83	5	0.17		
	Cor total	15.22	31			
	Model	16,709.07	20	835.45	10.37	0.0002 **

Note: ** indicates that the difference is extremely significant (*p* ≤ 0.01); * indicated significant difference (0.01 < *p* ≤ 0.05).

## Data Availability

The raw/processed data required to reproduce these findings cannot be shared at this time, as the data also form part of an ongoing study.
